# Collision tumor of pulmonary adenocarcinoma and small lymphocytic lymphoma: A rare case of concurrent malignancies in the same lymph node

**DOI:** 10.1016/j.ijscr.2024.110230

**Published:** 2024-09-03

**Authors:** Maaweya Jabareen, Abdalqader Aljaradat, Motaz Natsheh, Yousef Abu Asbeh, Hobaib Shawar

**Affiliations:** a Faculty of Medicine, Hebron University, Hebron, Palestine; b Al-Ahli hospital, Hebron, Palestine

**Keywords:** Collision tumor, Pulmonary adenocarcinoma, Small lymphocytic lymphoma, Lymphangitis carcinomatosis, Case report

## Abstract

**Background:**

Collision tumors, a rare and challenging diagnostic entity, are characterized by the simultaneous presence of two distinct histological neoplasms within the same anatomical site. The underlying mechanisms of collision tumors are not well understood, though various theories attempt to explain this phenomenon.

**Case presentation:**

A 77-year-old Palestinian man, a heavy smoker with multiple comorbidities, presented with a productive cough and significant weight loss. Computed tomography (CT) scan with IV contrast revealed extensive pulmonary involvement, mediastinal lymphadenopathy, and adrenal gland nodules. An excisional biopsy of a lymph node confirmed the presence of both metastatic pulmonary adenocarcinoma and small Lymphocytic Lymphoma (SLL) through histopathological and immunohistochemical analyses.

**Discussion:**

Collision tumors have been documented in various anatomical sites, such as the lung, gastrointestinal tract, skin, and genitourinary system. However, their occurrence in lymph nodes is exceptionally rare. Additionally, to our knowledge, a collision tumor involving both pulmonary adenocarcinoma and small lymphocytic lymphoma within the same lymph node has not been previously reported.

**Conclusion:**

Collision tumors are uncommon and should be considered in the differential diagnosis of complex oncological cases. Accurate diagnosis requires comprehensive investigations, including imaging studies and detailed pathological examinations.

## Highlights

• Collision tumors are neoplasms coexisting in some anatomical area.
• Pathogenesis of collision neoplasms is unknown and a matter of speculation.
• Collision tumor diagnosis is usually confirmed by pathology post-resection, as preoperative imaging often cannot provide a definitive diagnosis.
• The presence of two malignancies in one lymph node posed a significant challenge.

## Abbreviations

SLL: Small Lymphocytic Lymphoma
NHL: non-Hodgkin's lymphoma
BUN: Blood Urea Nitrogen
CT: Computed tomography
IV: Intravenous
PET: Positron Emission Tomography
TTF1: Thyroid Transcription Factor 1
PAX8: Paired Box Gene 8
CD: Cluster of Differentiation
SGPT: Alanine Aminotransferase
SGOT: Aspartate Aminotransferase
RBS: Random Blood Sugar
ALK PHOS: Alkaline Phosphatase
HCT: Hematocrit
Hb%: Hemoglobin
MCH: Mean Corpuscular Hemoglobin
MCHC: Mean Corpuscular Hemoglobin Concentration
MCV: Mean Corpuscular Volume
PLTS: Platelets
RBC: Red Blood Cells
WBC: White Blood Cells
H&E: Hematoxylin and Eosin

## 1 Introduction

This work has been reported in line with the SCARE criteria [1].

Collision tumors, characterized by the coexistence of two distinct histological neoplasms within the same anatomical site, a rare and challenging diagnostic entity in clinical practice. These tumors are marked by their unique histopathological features, where two different types of cancerous growths manifest independently within a shared tissue or organ without intermingling at the cellular level [[2], [3]].

The phenomenon of collision tumors remains poorly understood, and several hypotheses have been proposed to explain their occurrence. These include the “random collision effect,” where two independent neoplastic clones arise coincidentally within close proximity, and the “field cancerization” theory, which suggests that a common carcinogenic environment predisposes adjacent tissues to develop distinct neoplasms. Another proposed mechanism is “tumor-to-tumor carcinogenesis,” where one tumor influences the growth and development of a second neoplasm in its vicinity [4].

In recent literature, collision tumors have been reported in various anatomical sites, including the lung, gastrointestinal tract, skin and genitourinary system. However, their occurrence in lymph nodes remains exceptionally rare, particularly when involving metastatic adenocarcinoma from the lung and primary lymphoid malignancies [3].

We present a rare case of a collision tumor composed of metastatic adenocarcinoma and small lymphocytic lymphoma diagnosed and managed at a private healthcare center. This unique combination of two distinct neoplasms within the same anatomical site highlights the complexity and diagnostic challenge associated with collision tumors. Lung adenocarcinoma accounts for approximately 40 % of all lung cancers and is associated with a high mortality rate. It frequently metastasizes to the liver, adrenal glands, brain, and bones, and rarely to soft tissues [5]. Small lymphocytic lymphoma accounts for about 7 % of newly diagnosed non-Hodgkin's lymphoma (NHL) cases. Due to its rarity, only small series of SLL have been reported [6].

## 2 Case presentation

**Fig. 1:**
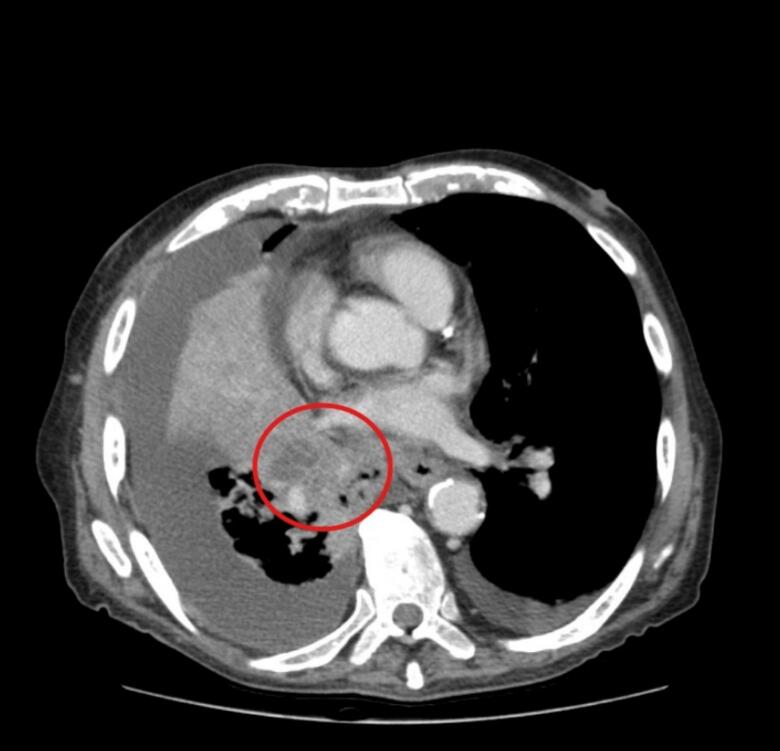
Axial CT scan with IV contrast showed ill-defined infiltrative hypodense right hilar perihilar lesion.

**Fig. 2:**
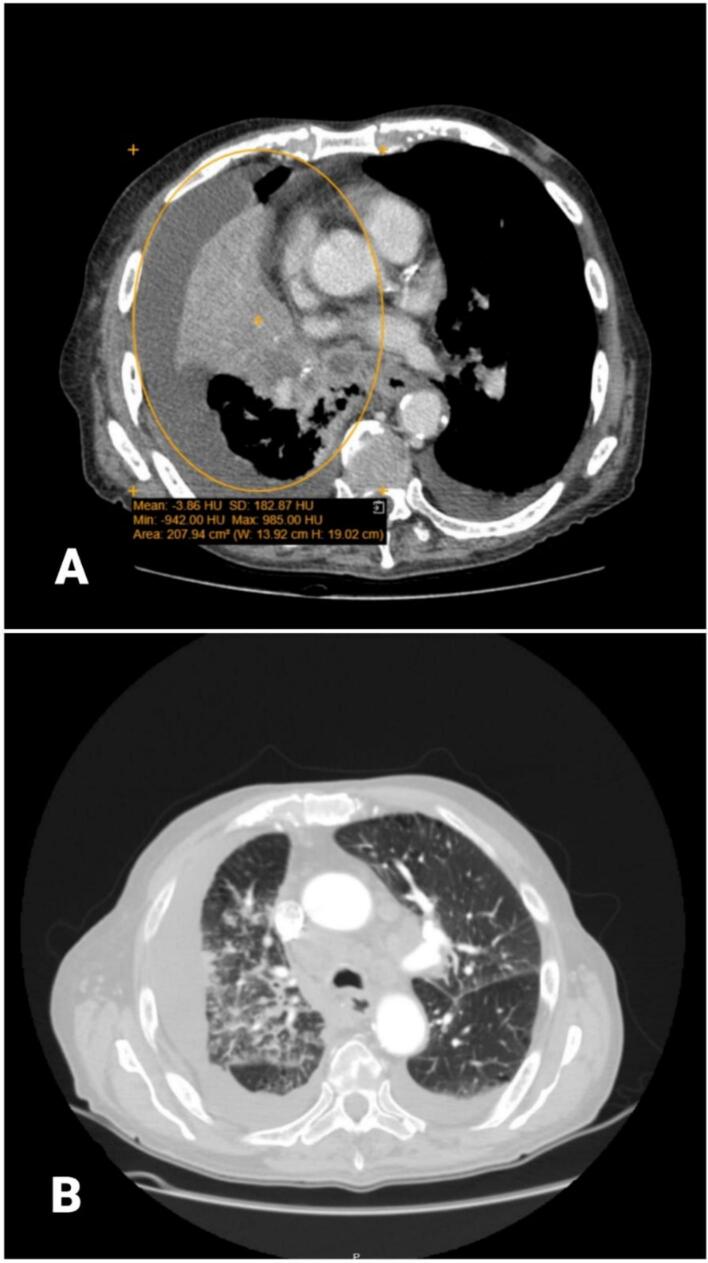
Axial CT scan with IV contrast showed A. Collapsed lung parenchyma in the right middle lobe and most of the right lower lobe. B. lymphangitis carcinomatosis.

A 77-year-old Palestinian man, a heavy smoker with a medical history of diabetes mellitus, hypertension, and benign prostatic hyperplasia, presented to the hospital with a 12-day history of productive cough and a notable 10 kg weight loss over the past 3 months. The cough, which had progressively worsened, was particularly severe at night and accompanied by exertional shortness of breath and chest pain. On examination, the patient appeared cachectic, and the respiratory assessment revealed reduced air entry on the right side. The laboratory investigations were performed and showed normocytic anemia, hyperglycemia, and a slight elevation in blood urea nitrogen (BUN) level. A chest x-ray showed consolidation of the right middle and lower lobes; a diagnostic bronchoscope was performed; and an external compression on the right bronchial tree was found without an endobronchial lesion. CT of the chest with IV contrast showed ill-defined infiltrative hypodense right hailer lesion infiltrate through the collapsed lung parenchyma (Fig. 1), encasing the superior lobar bronchus and bronchus intermedius, attenuating their lumen with almost complete obliteration and resultant subsequent collapse of the entire middle lobe and most of the basal segment of the right lower lobe (Fig. 2A), this mass also encases the right interlobar artery and the ipsilateral pulmonary veins, causing mild narrowing of their caliber while partially encasing the right upper lobe segment branching. The non-collapsed right lung field shows diffuse nodular septal thickening and peribronchovascular thickening with confluent patchy consolidation on top of the background of ground glass attenuation. These findings are in keeping with lymphangitis carcinomatosis (Fig. 2B). Left lung shows multiple nodules up to 1 cm in the left lower lobe, along with interlobular septal thickening and peribronchial thickening, mainly at the lower lobe, which is also suggestive of lymphangitis carcinomatosis.

**Fig. 3:**
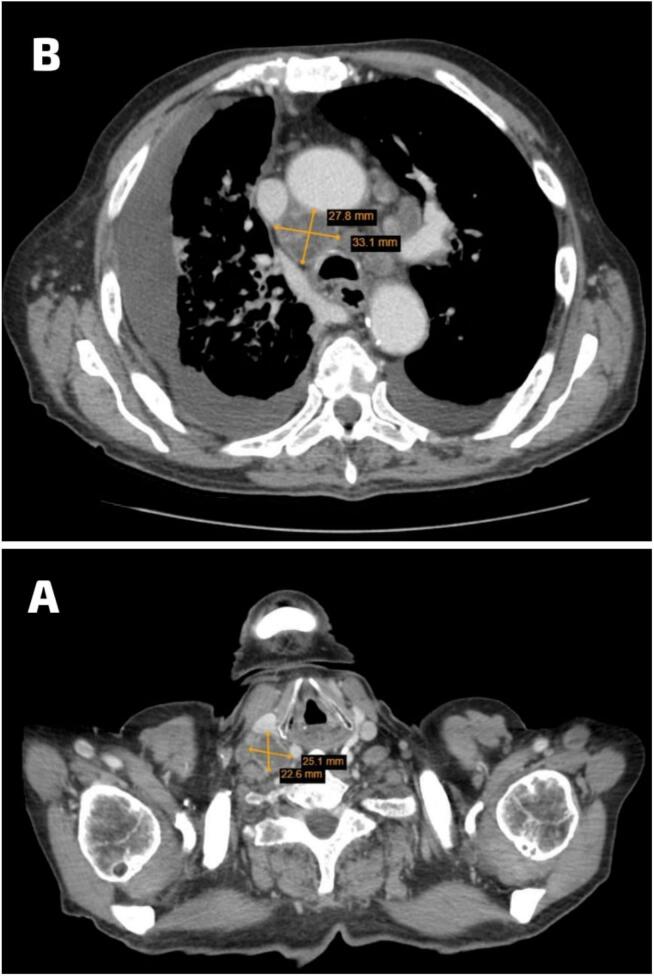
Axial CT scan with IV contrast showed A. multiple enlarged pathological lymph nodes in the right supraclavicular level B. Multiple enlarged mediastinal and hilar lymph nodes with central necrosis.

**Fig. 4:**
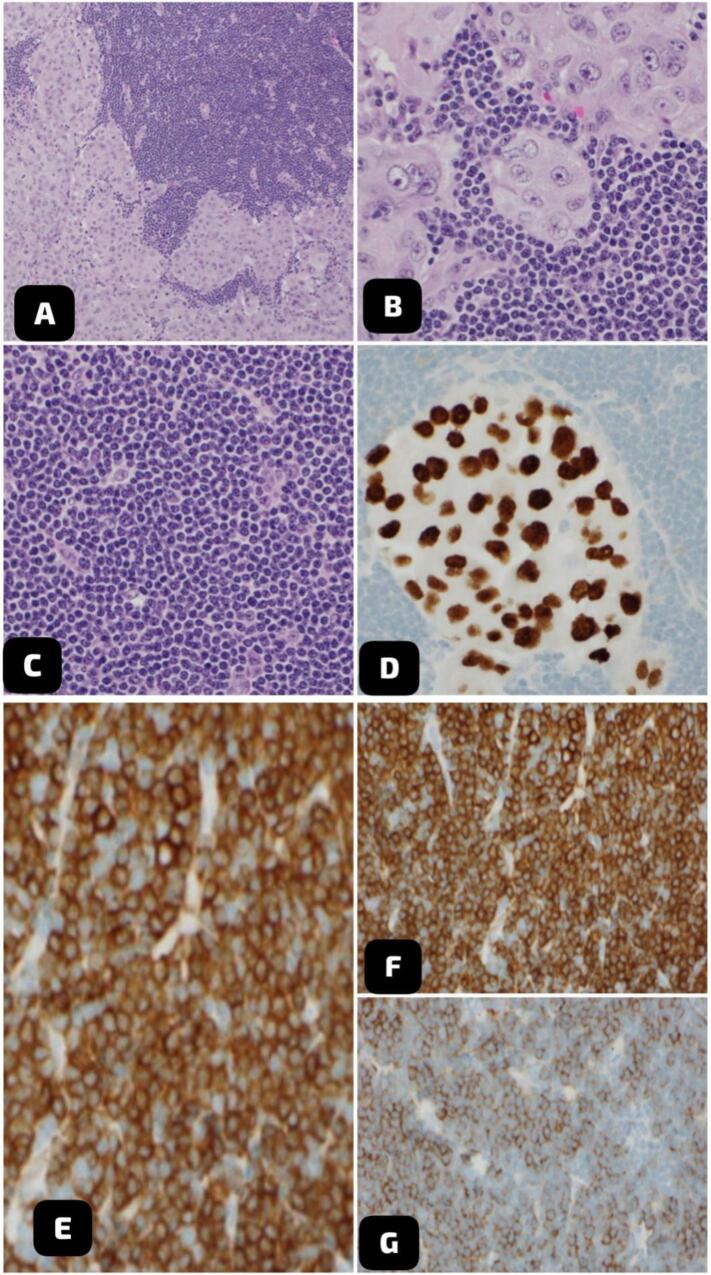
Histopathological findings of the biopsy specimens from the right cervical lymph node showed that the lymph nodes are involved by two malignant processes (collision tumors). A&B. Most of the lymph nodes are replaced by metastatic adenocarcinoma in a background of atypical small lymphocytes (H&E, 10×, & 20×). C. The atypical lymphocytes are monotonous, small, mature lymphocytes with soccer ball-like nuclei (H&E; 40×). D. TTF1 immunostain is positive in metastatic adenocarcinoma. E, F & G. CD20, CD5, and CD23 immunostains are positive in the atypical lymphocytes.

There are also multiple enlarged pathological mediastinal, hilar, right supraclavicular, and scanned lower cervical lymph nodes, most of them with peripheral enhancement and necrotic centers (Fig. 3A&B). Additionally, bilaterally enhanced adrenal nodules measuring up to 2 cm on the left and 1.4 cm on the right were also noted, concerning metastasis. Despite medical recommendations, the patient opted against undergoing a PET scan. The patient underwent a surgical excisional biopsy of the right lower cervical lymph nodes. Grossly, two lymph nodes were received, the largest measuring 1 × 1 × 1 cm. Histopathological examination revealed that the majority of lymph nodes are replaced by metastatic adenocarcinoma. The metastatic cells are positive for TTF1 and negative for PAX8 and P40 immunostains (Fig. 4). The residual lymphocytes are atypical, small, mature lymphocytes with soccer ball-like nuclei. These lymphocytes are positive for CD20, CD5, and CD23 immunostains and negative for CD3 immunostain (Fig. 4).

The patient was referred to an oncologist to discuss a proposed treatment plan. Despite the consultation, the patient ultimately declined both the consultation and any associated medications.

## 3 Discussion

A collision tumor is a rare phenomenon in which two distinct neoplasms occur concurrently in the same tissue or organ without intermingling at the histological level [7,8]. The occurrence of a collision tumor involving pulmonary adenocarcinoma and small lymphocytic lymphoma is an extremely rare event and, to our knowledge, has not been previously reported.

Lung cancer is the leading cause of cancer-related deaths worldwide. Prior to the 2015 WHO classification update, adenocarcinoma of the lung was diagnosed based on acinar or tubular patterns, mucin production, and positivity for TTF-1 and Napsin A. p40, the best marker for squamous cell carcinoma (SqCC) in terms of specificity, was only observed in about 3 % of adenocarcinoma cases [9].

Small lymphocytic lymphoma is a type of non-Hodgkin lymphoma characterized by the proliferation of small, mature lymphocytes primarily within the lymph nodes. Immunohistochemical staining shows that these lymphocytes are variably positive for CD20, weakly positive for CD5, and positive for CD23 [10].

Our case presents a 77-year-old male heavy smoker with a history of multiple comorbidities, presented with cough and significant weight loss. CT scan with IV contrast revealed a malignant-looking right lung lesion with its bulk in the hilar/perihilar region and features suggestive of lymphangitis carcinomatosis, along with pathological mediastinal, hilar, right supraclavicular, and lower cervical lymph nodes. A bilaterally enhanced adrenal nodule was also noted.

Histopathological examination of the cervical lymph nodes in this case confirmed metastatic adenocarcinoma, a finding consistent with the imaging findings and clinical suspicion. Immunohistochemical analysis showing positivity for TTF1 and negativity for PAX8 and P40 further supports the pulmonary origin of the metastasis; these metastatic cells coexist with atypical small mature lymphocytic cells, which were positive for CD20, CD5, and CD23.

Lymphangitic carcinomatosis, as observed in this case, is a rare form of cancer dissemination characterized by the presence of tumor emboli within the lymphatic vessels of the lungs. This leads to the development of diffuse interstitial pulmonary infiltrates. The rarity of this condition is underscored by the fact that only 65 studies were published on this topic between 2000 and 2018 [11,12], Patients afflicted with this rare cancer type frequently experience a challenging prognosis, primarily stemming from delayed diagnosis, underlying health conditions, and resistance to conventional therapies [12].

The concurrent diagnosis of small lymphocytic lymphoma, based on the histopathological examination of the cervical lymph nodes, adds a unique dimension to the case. To our knowledge, the combination of metastatic lung adenocarcinoma and small lymphocytic lymphoma in the same lymph node has not been previously documented.

Collision metastasis from lung cancer and malignant lymphoma are exceptionally rare, with only a few cases reported in the literature. For example, a 1998 study described a 71-year-old woman with a collision tumor consisting of squamous cell carcinoma and T-cell lymphoma [3]. Another case, reported in 2009, involved a 55-year-old man with a collision tumor featuring both adult T-cell leukemia/lymphoma and primary lung cancer [13].

Notably, the occurrence of collision metastasis from pulmonary adenocarcinoma and malignant lymphoma in the same lymph node is extremely rare [3,14]. However, in 2018, a study reported the first documented case of collision metastasis to the same lymph node from both pulmonary adenocarcinoma and Diffuse Large B-cell Lymphoma [14].

The treatment approach in cases such as ours is a comprehensive regimen incorporating surgery, chemotherapy, and radiation therapy. This approach is employed to effectively manage pain and alleviate related complications. However, the prognosis for the patient may be unfavorable due to the advanced stage of the disease [15].

In this case, the patient required a chest tube to manage worsening pleural effusion and was provided with palliative care. A consultation with an oncologist was recommended to optimize the management plan. Unfortunately, the patient refused the oncologist consultation and any related medications, and subsequent efforts to follow up with the patient and their family were unsuccessful. As a result, we are unable to know the ultimate prognosis or provide further care or support at this time.

## 4 Conclusion

This case presents a rare collision tumor involving both metastatic pulmonary adenocarcinoma and small lymphocytic lymphoma within the same lymph node in a 70-year-old Palestinian male. To our knowledge, this specific presentation has not been previously reported in the medical literature. The clinical complexity of this case highlights the importance of considering diverse differential diagnoses in complex oncological scenarios. Management challenges arise due to the advanced disease stage and the patient's refusal of treatment. Further research is needed to understand the underlying mechanisms of such collision tumors and to develop effective treatment strategies for similar complex malignancies.

## Informed consent

Written informed consent was obtained from the patient for publication of this case report and accompanying images. A copy of the written consent is available for review by the Editor-in-Chief of this journal on request.

## Ethical approval

Ethical approval was not applicable for this study, as our institution's IRB committee at Hebron University does not mandate approval for reporting individual cases or case series.

## Funding

This research did not receive any specific grants from funding agencies in the public, commercial, or not-for-profit sectors.

## Author contribution

M.A. handled conceptualization, data curation, and the writing of the original draft. A.A. writing: review, editing, and software. M.N. contributed to the investigation and visualization. H.S. managed resources and validation. Y.A. provided supervision.

## Guarantor

Maaweya Jabareen.

## Research registration number

N/A.

## Conflict of interest statement

None.

## Data Availability

All data supporting the study's findings are included in the article and are readily accessible.
